# Supplementary material for: FERN – a Java framework for stochastic simulation and evaluation of reaction networks
Source: BMC Bioinformatics. 2008 Aug 29;9:356. doi: 10.1186/1471-2105-9-356 (PMC2553347; doi:10.1186/1471-2105-9-356)
Supplement: Additional file 1 — FERN distribution, Version 1.3. This archive contains the FERN source code and binaries as well as documentation and example models in FernML and SBML. [file 1471-2105-9-356-S1.zip › fern/doc/javadoc/fern/tools/gnuplot/package-summary.html]

fern.tools.gnuplot


---


|  |  |  |
| --- | --- | --- |
| **Overview** \| **Package** \| Class \| **Use** \| **Tree** \| **Deprecated** \| **Index** \| **Help** | |  |
| **PREV PACKAGE**   NEXT PACKAGE | **FRAMES**    **NO FRAMES**     **All Classes** |  |


---

## Package fern.tools.gnuplot

Provides classes to use gnuplot from java.

**See:**
  
          **Description**

| **Class Summary** | |
| --- | --- |
| **ArrayMatrixAxes** | Implementation of `Axes` to use with array matrixes of any number type (the type of the arrays is inferred by using the reflection api). |
| **Axes** | Contains the data columns for `GnuPlot`. |
| **CollectionAxes** | Implementation of `Axes` to use with `Collection`s or `Iterable`s of any number type or array of numbers (the type of the collection is inferred by using the reflection api). |
| **GnuPlot** | The class `GnuPlot` provides methods for handling gnuplot data. |
| **TransposedArrayMatrixAxes** | Implementation of `Axes` to use with transposed array matrixes of any number type (the type of the arrays is inferred by using the reflection api). |

## Package fern.tools.gnuplot Description

Provides classes to use gnuplot from java.

---


|  |  |  |
| --- | --- | --- |
| **Overview** \| **Package** \| Class \| **Use** \| **Tree** \| **Deprecated** \| **Index** \| **Help** | |  |
| **PREV PACKAGE**   NEXT PACKAGE | **FRAMES**    **NO FRAMES**     **All Classes** |  |


---
